# Reovirus enhances cytotoxicity of natural killer cells against colorectal cancer via TLR3 pathway

**DOI:** 10.1186/s12967-021-02853-y

**Published:** 2021-05-01

**Authors:** Shiqi Long, Yangzhuo Gu, Yuanyuan An, Xiaojin Lin, Xiaoqing Chen, Xianyao Wang, Chunxiang Liao, Weiwei Ouyang, Nianxue Wang, Zhixu He, Xing Zhao

**Affiliations:** 1 Center for Stem Cell and Tissue Engineering Research/School of Basic Medical Sciences, Guizhou Medical University, Guiyang, 550004 Guizhou China; 2 Key Laboratory of Adult Stem Cell Transformation Research, Guiyang, 550004 China; 3 State Key Laboratory of Biotherapy and Cancer Center, West China Hospital, Sichuan University, and Collaborative Innovation Center for Biotherapy, Chengdu, 610041 China; 4 Department of Oncology, The Affiliated Hospital of Guizhou Medical University and Guizhou Cancer Hospital, Guiyang, 550004 China; 5 Department of Pediatrics, Affiliated Hospital of Zunyi Medical University, Zunyi, 563000 Guizhou China

**Keywords:** Natural killer, Reovirus, Colorectal cancer, Antibody-dependent cell-mediated cytotoxicity, Toll-like receptor 3

## Abstract

**Background:**

Cetuximab has been approved for use for first-line treatment of patients with wild-type *KRAS* metastatic colorectal cancer (CRC). However, treatment with cetuximab has shown limited efficacy as a CRC monotherapy. In addition, natural killer (NK) cell function is known to be severely attenuated in cancer patients. The goal of this study was to develop a new strategy to enhance antibody-dependent cell-mediated cytotoxicity (ADCC) mediated by NK cells, in combination with cetuximab against CRC cells.

**Methods:**

Ex vivo expanded NK cells were stimulated with reovirus, and reovirus-activated NK cells mediated ADCC assay were performed on CRC cells in combination with cetuximab. The synergistic antitumor effects of reovirus-activated NK cells and cetuximab were tested on DLD-1 tumor-bearing mice. Finally, Toll-like receptor 3 (TLR3) knockdown in NK cells, along with chemical blockade of TLR3/dsRNA complex, and inhibition of the TLR3 downstream signaling pathway, were performed to explore the mechanisms by which reovirus enhances NK cell cytotoxicity.

**Results:**

We first confirmed that exposure of NK cells to reovirus enhanced their cytotoxicity in a dose-dependent manner.We then investigated whether reovirus-activated NK cells exposed to cetuximab-bound CRC cells exhibited greater anti-tumor efficacy than either monotherapy. Co-culture of CRC cell lines with reovirus-activated NK cells indicated that NK cytotoxicity was significantly higher in combination with cetuximab, regardless of *KRAS* mutation status or EGFR expression level. We also found that reovirus activation of NK cells, in conjunction with cetuximab, resulted in significantly stronger anti-tumor efficacy.Finally, TLR3 knockdown, inhibition of TLR3/dsRNA complex or TBK1/IKKε demonstrated that activation of NK cells by reovirus was dependent on TLR3 and its downstream signaling pathway.

**Conclusions:**

This study demonstrated that combination treatment of reovirus-activated NK cells with cetuximab synergistically enhances their anti-tumor cytotoxicity, suggesting a strong candidate strategy for clinical treatment of CRC.

**Supplementary Information:**

The online version contains supplementary material available at 10.1186/s12967-021-02853-y.

## Background

Colorectal cancer (CRC) is the third most common cancer and the fourth leading cause of cancer-related death worldwide [1, 2]. In the past two decades, significant advances have been made in the treatment of CRC, including the introduction of oxiplatin, irinotecan, and targeted therapeutic monoclonal antibodies (mAb) like cetuximab [3, 4].Cetuximab, a chimeric immunoglobulin G1(IgG1) mAb that targets the epidermal growth factor receptor (EGFR), was approved for use in combination with FOLFIRI for first-line treatment of patients with wild-type *KRAS* metastatic CRC [5].Cetuximab specifically binds to EGFR,to block the endogenous EGFR ligands and consequently disrupt EGFR-driven signaling, leading to cell cycle arrest and apoptosis. In addition to cetuximab’s direct anti-tumor effects, in vivo evidence from both murine models and clinical series suggest that cetuximab exerts antitumor effects in part due to antibody-dependent cell-mediated cytotoxicity (ADCC) [6, 7]. ADCC is a mechanism of innate effector cell immunity, initiated by the binding of receptors for the Fc domain of IgG (FcγRs).FcγRs and their subclasses are expressed on dendritic cells (DCs), monocytes, macrophages, and natural killer (NK) cells. For NK cells, recognition of IgG1 mAbs by FcγRIIIa (CD16) results in enhanced NK cell degranulation, cytokine secretion, and cytotoxicity [8, 9].However, despite cetuximab’s multiple mechanisms of action, responses to cetuximab are limited: only 1 in 5 patients with metastatic CRC responds to cetuximab [10].New approaches are therefore needed to enhance its efficacy. To this end, increasing the NK cell response to cetuximab therapy could potentially enhance the effects of ADCC in CRC therapy.

NK cells are a crucial target for cancer immunotherapy since they can directly kill tumor cells without prior sensitization or major histocompatibility complex (MHC) restriction. NK cells recognize tumor cells via activating receptors and inhibitory receptors. Activated NK cells directly kill tumor or virus-infected cells through release of cytotoxic granules and proinflammatory cytokines. Recent finding indicate that NK cells exhibit decreased activity, reduction in the proportion of IFN-γ secreting NK cells,and predominant CD16^dim/neg^ subpopulation in cancer patients [11, 12]. Additionally, multiple reports found that NK cell function in cancer patients is severely attenuated [13]. Increased expression of programmed cell death protein 1(PD-1) or T cell immunoglobulin and mucin domain-containing molecule-3(Tim-3) on NK cells suppress NK cell cytotoxicity and correlate with poor prognosis [14–16]. Therefore, extensive studies have investigated different strategies to enhance NK cell cytotoxicity and thereby improve the clinical outcomes of NK cell-based immunotherapies [17–19].

Here, in this work, we sought to develop a new strategy to enhance NK cell cytotoxicity while also improving the effects of cetuximab-mediated ADCC. Our previous research found that reovirus can directly activate fresh NK cells in vitro, and that reovirus-loaded NK cells could deliver reovirus to tumor cells in the presence of neutralizing antibodies (NABs) [20, 21]. Therefore, we hypothesized that the antitumor efficacy of cetuximab could be improved by combination with reovirus activation of NK cells. In this strategy, NK cells are first activated with reovirus, followed by exposure of the reovirus-activated NK cells to cetuximab-bound CRC cells. Using multiple CRC cell lines with different *KRAS* mutation status and EGFR expression levels we found that reovirus directly enhanced NK cell cytotoxicity,and that, in combination with cetuximab, reovirus-activated NK cells exhibited increased CRC cell killing in vitro*,*regardless of *KRAS* mutation or EGFR expression.Then using human colorectal tumor xenograft models, we also observed that reovirus activation of NK cells, in conjunction with cetuximab, provided significantly greater anti-tumor effects than either monotherapy. Finally, using Toll-like receptor 3 (TLR3) knockdown NK cells,chemical blockade of TLR3/double-stranded RNA (dsRNA) complex,and chemical inhibition of the TLR3 downstream pathway, we further determined that reovirus activation of NK cells is mediated in a TLR3 signaling pathway-dependent manner.These findings of synergistically enhanced NK cell anti-tumor activity provide a viable framework for improvement of clinical strategies against CRC.

## Materials and methods

### Cells, virus, and reagents

The murine fibroblastic cell line L929, the EGFR-expression CRC cell lines DLD-1 (*KRAS*-mutant, EGFR medium), Caco-2 (*KRAS*-WT, EGFR high), and HT-29 (*KRAS*-WT, EGFR low) were obtained from the China Center for Type Culture Collection (CCTCC) [6]. All cell lines were cultured in MEM and RPMI-1640 (Hyclone) supplemented with 10% fetal bovine serum (FBS, Gibco), 1% (v/v) glutamine (Gibco) and 1% (v/v) penicillin/streptomycin (Hyclone). All cells were maintained at 37 °C, 5% CO2 in a humidified atmosphere.Reovirus type 3 Dearing strain was obtained from ATCC (VR-824). Reovirus was propagated in L929 cells, and titrated with a standard plaque assay protocol for evaluating L929 cells. To generate UV-inactivated reovirus. Reovirus in PBS were exposed to UV (shortwave 254 nm) for 30 min. The UV-induced loss of reoviral replicability was confirmed with a L929 cell viability assay.Live and UV-inactivated reovirus were stored at -80 °C until use. Cetuximab (murine-human chimeric anti-EGFR, IgG1) was obtained from Bristol-Myers Squibb. Control IgG1 purchased from Sigma-Aldrich.Bx795 (Sigma-Aldrich) was used to inhibit TBK/IKKε,TLR3/dsRNA complex inhibitor was purchased from Calbiochem. Polyinosinic:polycytidylic acid [Poly (I:C)] HMW was obtained from InvivoGen.

### Ex vivo NK cell expansion

Peripheral blood mononuclear cells (PBMCs) from healthy donor blood were isolated by means of standard density gradient centrifugation with the use of Ficoll-Paque Plus (GE healthcare).NK cells were enriched from PBMCs as mentioned below. Briefly, 5 × 10^6^ PBMCs were cultured in GT-T551 H3 medium (Takara Bio Inc.) supplemented with 1% autologous serum and 200 IU/mL of rhIL-2 (PeproTech), and co-cultured with 5 × 10^6^ irradiated inactivated K562-mbIL-21 feeder cells (Lifeark). Media were changed every 3–4 days with a weekly addition of K562-mbIL-21.NK cells were harvested after 14–16 days of incubation and assessed for purity (NK cells defined as CD3^−^CD14^−^CD56^+^) via flow cytometry (> 95% purity was achieved). NK cells were aliquoted and cryopreserved in liquid nitrogen until use. To minimize phenotypic changes, all NK cells were used within two months of the initial freezing date.

### Cytotoxicity assay

The cytotoxic activity of NK cells was assessed using Cell Counting Kit-8 (CCK-8, Dojindo).Briefly, NK cells were thawed in a 37 °C water bath and washed twice in GT-T551 H3 medium before use. NK cells were maintained in GT-T551 H3 medium for at least 2 h, before they were resuspended at 2 × 10^6^ cells/mL and incubated with 10 multiplicity of infection (MOI) reovirus or 1 μg/mL Poly(I:C) complexed with Lipofectamine 3000 (Invitrogen) for 12 h at 37 °C and 5% CO2. After 12 h of pretreatment, NK cells were incubated with target tumor cells (DLD-1, Caco-2, and HT-29) at 5:1 E:T ratios for 4 h in U-bottom 96-well plates at 37 °C, 5% CO2. For ADCC assay, target tumor cells were incubated with 1 μg/mL cetuximab or control antibody IgG1 for 1 h. Following incubation, the cytotoxicity assay was performed. For some blocking experiments, reovirus or Poly(I:C) activated NK cells were treated with 10 μM Bx795 or 10 μM TLR3/dsRNA complex inhibitor for 12 h at 37 °C and 5% CO2 prior to the addition of NK cells. After a 4-h incubation, 10 μL of supernatant was collected from each well and the absorbance at 450 nm of each well was measured according to the manufacturer’s instructions. All experiments were done in triplicate.

### siRNA-mediated gene knockdown

To knock down the expression of TLR3, NK cells were transfected with siRNA against TLR3 (siTLR3 no. 337 5′-GCUUGGAUGUAGGAUUUAATT-3′ & 5′-UUAAAUCCUACAUCCAAGCTT-3′, Gene Pharma) or random control siRNA (5′-UUCUCCGAACGUGUCACGUTT-3'. & 5′-ACGUGACACGUUCGGAGAATT-3′, Gene Pharma) using the Lipofectamine 3000 transfection reagent (Invitrogen, USA) for the indicated durations. The TLR3 knockdown efficacy was confirmed by qPCR and Western Blotting analysis. Following a 48-h incubation, NK cells were incubated with 10 MOI reovirus or 1 μg/mL Poly(I:C) for 12 h prior to coculture with DLD-1 cells.

### Western blotting

Cell lysates were prepared in cell lysis buffer. Extracts were analyzed by SDS-PAGE (Invitrogen) and western blot. The following antibodies were used at specified concentrations for immunoblots:TLR3 (Clone D10F10; 1:1,000; Cell Signaling Technology; #6961) and β-actin (Clone 5B7; 1:5,000;ImmunoWay; YM3028). The secondary antibody was HRP-conjugated donkey anti-rabbit IgG (H + L) (1:10,000; Biodragon; BF03008X). Antigen–antibody complexes were visualized by enhanced chemiluminescence (Bio-Rad). Western blots were quantified using ImageJ software (National Institutes of Health).

### Real-time quantitative PCR (qPCR)

Total RNA was extracted from cells using Trizol reagent (TaKaRa) and reverse-transcribed into cDNA using iScript cDNA Synthesis Kit (BioRad). The mRNA expression of GZMH, GZMM,PRF1,TNF, TLR3, T3D, and β-actin was quantified by SYBR Green real-time PCR. Real-time PCR amplifications were performed using Premix Ex TaqTM (TaKaRa) on a Bio-Rad CFX96 system. The PCR primers used in this study (Additional file 1: Table S1) were purchased from Sangon Biotech (Shanghai) Co., Ltd. Amplifications were performed by activation of Hot Start DNA polymerase at 95 °C for 30 s, followed by 40 cycles at 95 °C for 5 s and 60 °C for 45 s. Data were calculated using Bio-Rad CFX manager software v.2.1 and the ΔΔCT method, and expressed as relative quantities after β-actin normalization.

### ELISA

For certain experiments, the supernatants were collected and stored at − 80 °C until the time of use. The levels of IFN-γ, TNF-α, and perforin in supernatants were determined with the Human IFN-gamma Quantikine ELISA Kit (R&D Systems), the Human TNF-alpha Quantikine ELISA Kit (R&D Systems) and the Perforin human ELISA Kit (Abcam) according to the manufacturers’ protocols.

### Tumor transplantation and therapy

All animal experiments performed during this study were governed by the approval of the Ethics Committee at Guizhou Medical University, Guiyang, Guizhou Province, China. Five-to six-week-old female athymic BALB/c *nu/nu* mice were obtained from Beijing HFK Bio-Technology Co., Ltd and maintained at the Animal Experimental Center of Guizhou Medical University. DLD-1 cells (2 × 10^6^) were implanted s.c. on the left flank. After tumors reached ~ 50 mm^3^, mice were divided into four groups on day 10. Mice received an intravenous injection of phosphate-buffered saline (PBS, 100 μL), or NK cells pretreated with 10 MOI reovirus monotherapy (Reo-NK, 1 × 10^7^ cells), or were intraperitoneally treated with cetuximab at 200 μg monotherapy, or sequential intravenous injection of 1 × 10^7^ Reo-NK 12 h prior to being intraperitoneally treated with cetuximab dosed as in the monotherapy group. Animals were dosed for 3 weeks. Tumor size was measured with a caliper twice a week and calculated as length × width × height. Mice were sacrificed at day 26. This in vivo model was piloted with three mice per group and repeated with five mice per group. Tumors, liver and kidney were collected from each mouse, and then the tumors were used for qPCR analysis. The section of liver and kidney were stained by Hematoxylin and Eosin(H&E) according to manufacturer’s instruction.

### Flow cytometry immunophenotyping

For NK cell staining, we used the following antibodies: anti-CD3 (clone: UCHT1), anti-CD56 (clone:MEM-188), anti-CD69 (clone: FN50) all from BioLegend; and anti-TLR3 (clone: TLR3.7, Invitrogen). FITC-IgG2b, κ isotype (BD Biosciences) was used as an isotype control. All cell samples were pre-incubated with anti-CD16/32 antibody to block non-specific binding. Following Fc-receptor blocking. Cells were washed once with cold FACS buffer (PBS supplemented with 2% FBS and 0.1% sodium azide). Cells were stained 30 min at room temperature with the surface-staining antibodies.TLR-3 intracellular staining was performed using the Cytofix/Cytoperm Kit (BD Biosciences). Stained cells were collected on a FC500 flow cytometer (Beckman Coulter Inc.), and data were analyzed using Flowjo software.

### Statistical analyses

Statistical analyses were performed using GraphPad Prism version 8 software. For normally distributed variables, comparisons between two groups were performed by the parametric Student's t-test; multiple group comparisons were carried out by one-way analysis of variance (ANOVA). Significant differences were defined as: * *p* < 0.05, ** *p* < 0.01, *** *p* < 0.001.

## Results

### Stimulation with reovirus enhances NK cell-mediated cytotoxicity

**Fig. 1:**
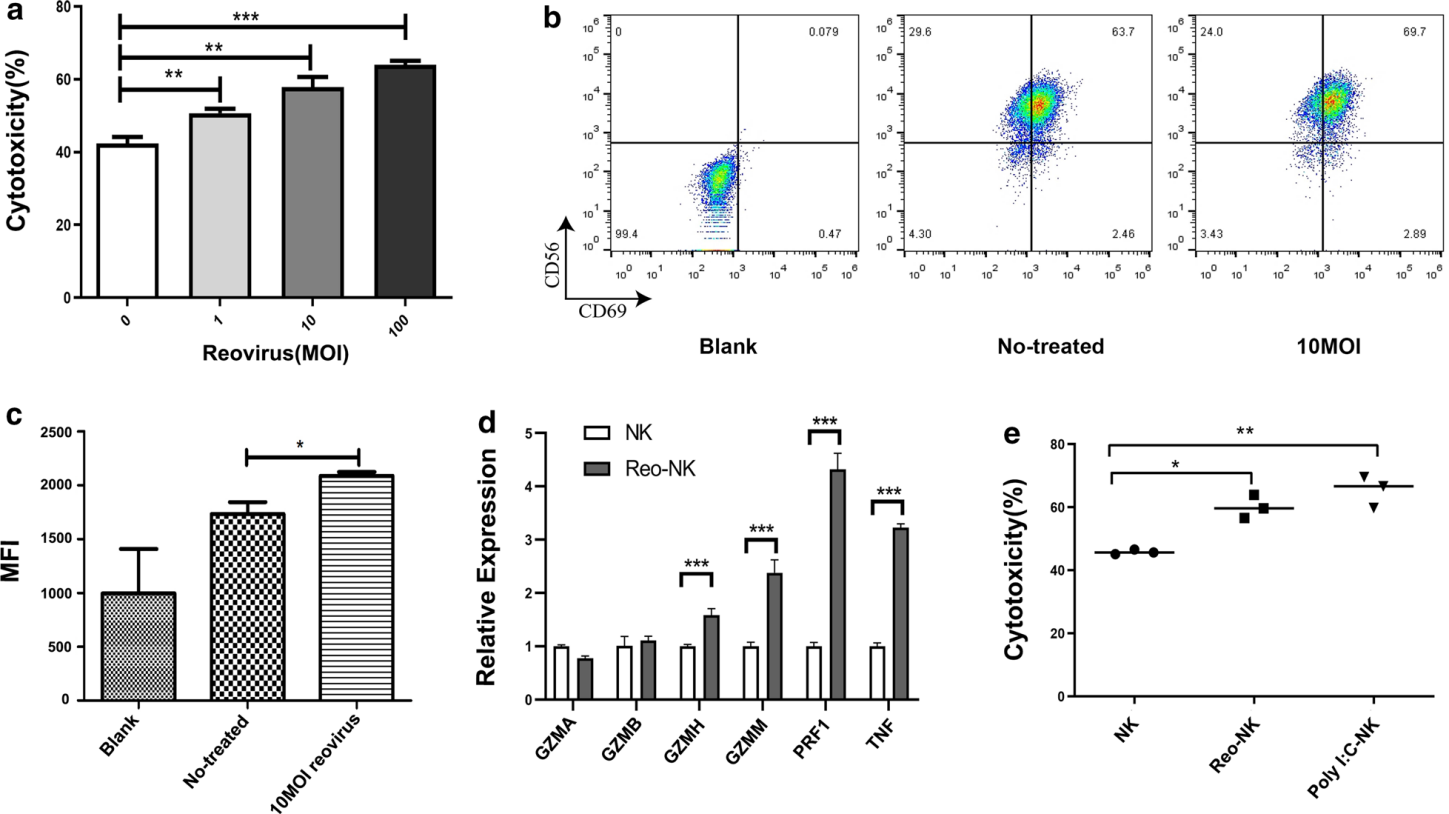
Effects of reovirus on NK cells. **a** NK cells were incubated in the absence or presence of 1, 10, or 100 MOI reovirus at 37℃ for 12 h. DLD-1 cells were used as target cells. Cytotoxic activity was measured at 4 h by CCK-8 assays performed on target cells at an E:T ratio of 5:1. All samples were assayed in triplicate and these data are representative of three independent experiments.(**p < 0.01, ***p < 0.001). **b** Representative flow cytometry plots showing the expression of CD69 on NK cells stimulated or not with 10 MOI reovirus for 12 h. **c** Compiled data (mean ± SEM)from three independent experiments showing mean fluorescence intensity (MFI) for CD69, **d** qPCR evaluation of the relative expression of GZMA, GZMB, GZMH, GZMM, PRF1, and TNF in NK cells after 12 h exposure to 10 MOI reovirus or medium alone (control; NK).qPCR experiments were performed twice with three technical replicates per sample. Data are means ± SD (***p < 0.001). **e** NK cells were incubated at 1 × 10^6^ cells/mL for 12 h in medium alone or 10 MOI reovirus; NK cells were transfected with 1 μg/mL Poly(I:C) by Lipofectamine and also cultured for 12 h. The NK cells stimulated under the various conditions were then co-cultured with DLD-1 cells for 4 h at a 5:1 E:T ratio. NK cell cytotoxicity was determined by CCK-8 assay. Data shown above are from a representative assay selected from three independent experiments. (*p < 0.05, **p < 0.01)

Given previous findings that NK cells can lyse tumor cells without prior exposure, and that NK cell cytotoxicity can be enhanced through pre-treatment with Poly(I:C), IL-2, or IL-15 [22, 23], we therefore used ex vivo expanded NK cells to evaluate their cytotoxicity following reovirus mediated activation.The results of NK cell cytotoxicity analysis showed that stimulation with different titers of reovirus and subsequent co-culture with DLD-1 target cells at a 5:1 effector-to-target-cell (E:T) ratio enhanced NK cell cytotoxicity in a dose-dependent manner (Fig. 1a). As CD69 is a marker for NK cell activation, we also assessed their activation through flow cytometry assays, which confirmed that CD69 was up-regulated in response to reovirus stimulation (Fig. 1b, c).Perforin (PRF1) and granzymes (such as GZMA, GZMB, GZMM and GZMH) represent two distinct categories of cytotoxic proteins released by NK cells. In addition, TNF-α has been shown to work in concert with IFN-γ to promote NK cell cytotoxicity. We applied qPCR analysis, which showed that the expression of GZMH, GZMM, PRF1, and TNF were all up-regulated in the NK cells treated with 10 MOI of reovirus for 12 h (Fig. 1d).

Poly(I:C), a synthetic ligand mimicking viral dsRNA, was shown in previous studies to increase the cytotoxicity of NK cells [24, 25]. In light of these findings, we next tested the effects of lipofectamine-packaged Poly(I:C) on NK cells. NK cells were treated with 10 MOI reovirus or 1 μg/mL Poly(I:C) for 12 h prior to co-culture with DLD-1 cells. We found that the cytotoxicity of NK cells was significantly increased following treatment with 10 MOI reovirus (60.10 ± 3.67, n = 3,*p* < 0.05) or lipofectamine-Poly(I:C) (65.38 ± 5.05, n = 3, *p* < 0.01) compared to that of un-activated control cells (45.84 ± 0.72, n = 3) (Fig. 1e). As reovirus has a double stranded RNA genome, which suggests that NK cells are activated by reovirus in a manner similar to that of Poly(I:C). Taken together, these results indicate that NK cell cytotoxicity can be enhanced through exposure to reovirus.

### Stimulation reovirus-activated NK cells enhance cetuximab-mediated ADCC against CRC cells

**Fig. 2:**
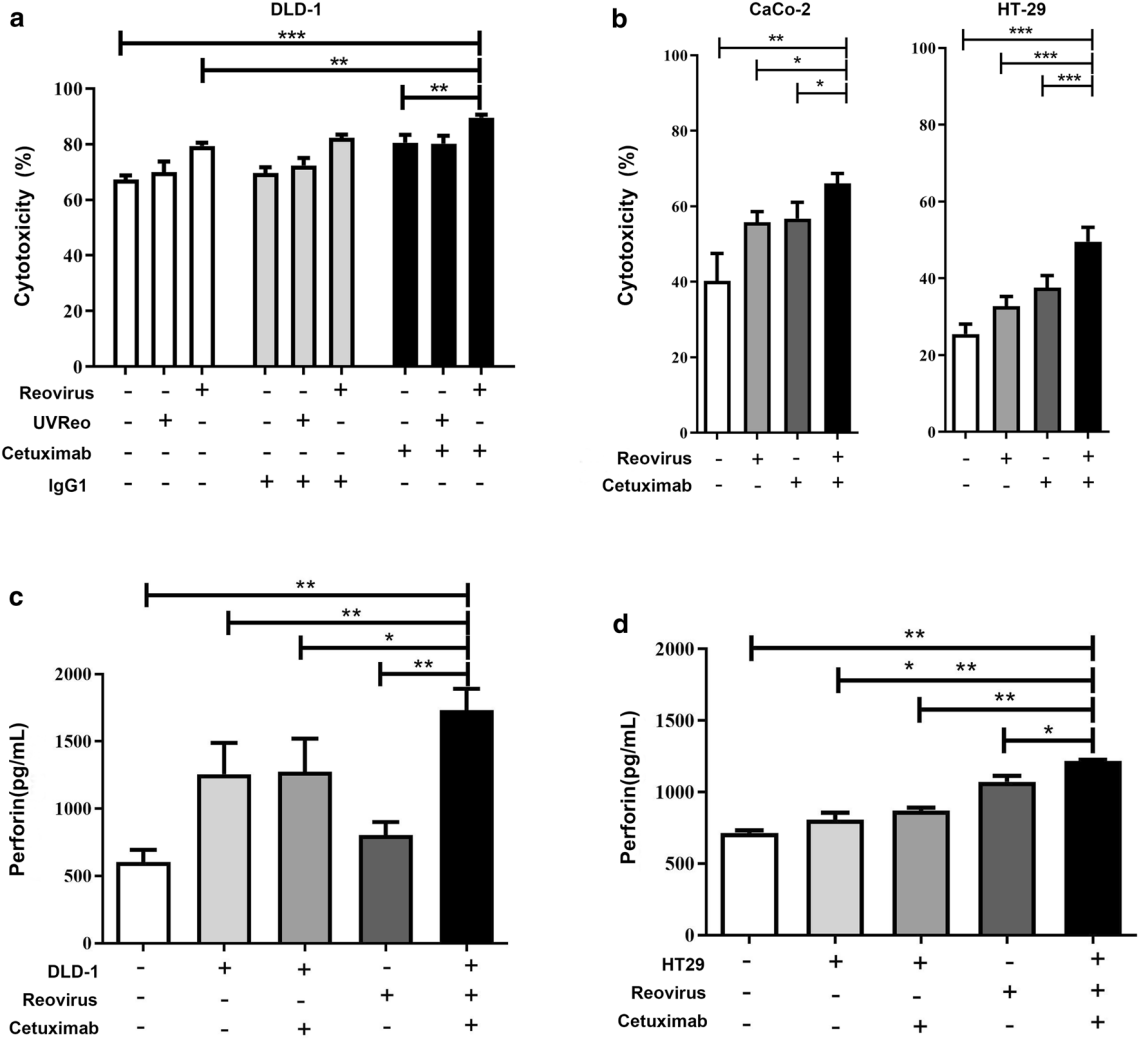
Reovirus-activated NK cells exhibit enhanced ADCC-mediated killing of colorectal cancer cells in combination with cetuximab. NK cells, as effectors, were activated with 10 MOI reovirus or UV-inactivated reovirus at 37℃ for 12 h. **a** DLD-1, or **b** Caco-2 and HT29 cells were used as target cells. Cetuximab and isotype control IgG1 were applied at 1 μg/mL and cytotoxic activity was measured by CCK-8 assay at an E:T = 5:1 ratio after 4 h of co-culture. *p < 0.05, **p < 0.01, ***p < 0.001. Data represent means ± SD from at least 3 independent experiments. **c**, **d** Levels of perforin secretion from reovirus-activated NK cells against DLD-1 or HT29 combined with cetuximab were determined by ELISA

Previous studies have reported that cetuximab can be used as a bridge to connect NK cells and CRC cells to trigger ADCC, thus killing colorectal cells [8, 26]. Therefore, promoting NK cytotoxicity can enhance the effects of ADCC mediated by cetuximab. Moreover, the ability of reovirus to enhance NK cytotoxicity makes it an ideal candidate for combination therapeutic strategies, such as with cetuximab.To examine whether reovirus activation of NK cells also enhances ADCC against EGFR-expressing CRC cells, we used ex vivo expanded NK cells activated with 10 MOI reovirus for 12 h to perform ADCC assays on colorectal cancer cells also treated with cetuximab. DLD-1 (*KRAS*-mutant, EGFR medium), Caco-2 (*KRAS*-WT, EGFR high), and HT-29 (*KRAS*-WT, EGFR low) were incubated with NK cells at a 5:1 E:T ratio [6], and we found that NK cells could efficiently kill DLD-1 (Fig. 2a), as well as Caco-2 and HT-29 (Fig. 2b) target cells. Furthermore,NK cell cytotoxicity was increased after activation with 10 MOI reovirus, and the reovirus-activated NK cells showed enhanced ADCC in combination with cetuximab treatment, indicated by decreasing tumor cell viability. Colorectal cancer cells were all highly sensitive to killing by reovirus-activated NK cells regardless of *KRAS* genotype or EGFR expression level, and reovirus further enhanced NK-cell cytotoxicity against cetuximab-coated colorectal cancer cells (Fig. 2a, b).

As NK cell cytotoxicity is mediated by the main effector molecule perforin, which creates pores on the target cells to facilitate entry of apoptosis-inducing granzymes, we therefore hypothesized that one mechanism by which reovirus might enhance and prime NK cell function is by increasing perforin release from NK cells. Detection by ELISA assays supported this hypothesis, and indeed, reovirus-activated NK cells released significantly higher levels of perforin with cetuximab against DLD-1 and HT-29 cells (Fig. 2c and d). Taken together, these data indicate that stimulation with reovirus enhances NK cell-mediated ADCC against colorectal cancer cells, and synergistically so in combination treatment with cetuximab.

### Reovirus-activated NK cells enhance the antitumor activity of cetuximab against KRAS-mutant tumors in vivo

**Fig. 3:**
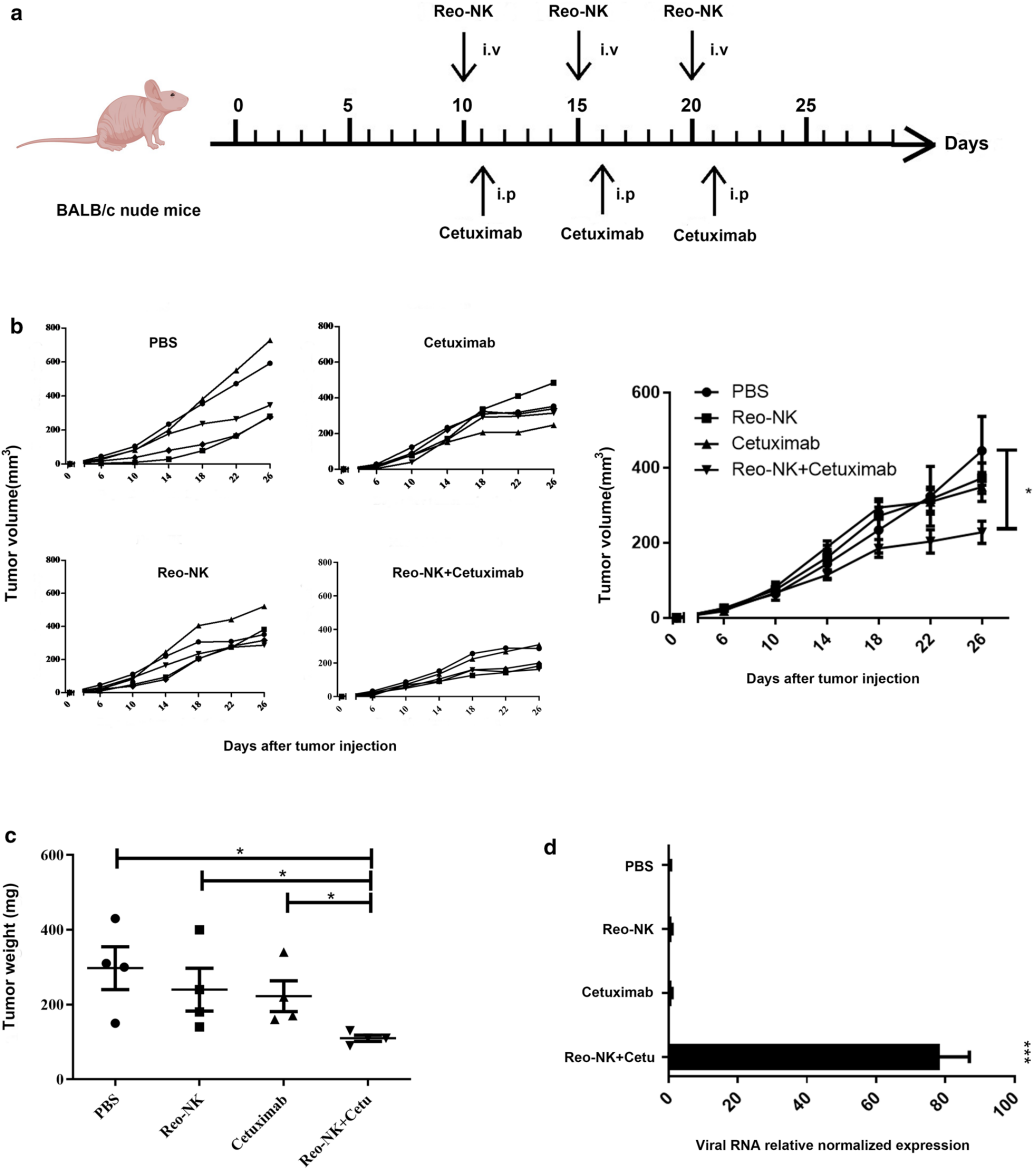
In vivo synergistic antitumor effects of reovirus-activated NK cells and cetuximab in DLD-1 tumor-bearing mice. BALB/c nude mice were injected subcutaneously with 2 × 10^6^ DLD-1 cells. **a** On day 10 after tumor inoculation, mice inoculated with DLD-1 then received intravenous injection with either PBS or 1 × 10^7^ reovirus-activated NK cells (Reo-NK) or 200 μg intraperitoneal cetuximab monotherapy on day 11, or sequential intravenous injection of 1 × 10^7^ Reo-NK 12 h prior to intraperitoneal cetuximab dosed as in the monotherapy group. Each injection was repeated weekly for a total of 3 injections (n = 5 per group). **b** Tumor volume was monitored over time and mean tumor volumes ± SD were calculated for each group (right). *p < 0.05 compared with control group. **c** Tumor weight of each group. *p < 0.05. **d** qPCR analysis of the relative T3D reovirus gene expression levels in tumor tissues. The *β-actin* gene was used as an internal reference. Relative expression was calculated using the ΔΔCt method. Data represent means ± SEM (n = 3). ***p < 0.001

Since the combination of cetuximab with reovirus-activated NK cells together led to increased lysis of colorectal cancer cells in vitro, we next assessed the efficacy of this combination therapy using human colorectal tumor xenograft models. Expanded NK cells were activated with 10 MOI reovirus (Reo-NK), followed by transfer into tumor-bearing mice,or intraperitoneal treatment with 200 μg cetuximab per mouse 1 day after transfer, or a combination of reovirus-activated NK cells and cetuximab. Animals were dosed for three weeks (Fig. 3a). The xenograft tumor growth curves showed restriction in tumor growth evidently following the third treatment. By day 26, we observed that the combination therapy significantly suppressed tumor growth(227.8 ± 65.76 mm^3^, *p* < 0.05), although reovirus- activated NK cells (371.9 ± 91.12mm^3^) and cetuximab (348.1 ± 86.30 mm^3^) also both showed inhibitory effects on tumor growth (Fig. 3b) compared with the PBS control (444.8 ± 204.1 mm^3^). We also evaluated the tumor weight for each group, and the results confirmed that the combination therapy significantly inhibited tumor growth in nude mice over that of individual treatments (Fig. 3c). Given that Reo-NK and cetuximab combined therapy resulted in strong antitumor effects against localized DLD-1 tumors, we next sought to determine whether the efficacy was related to tumor cell lysis by the reovirus. qPCR results showed significantly detectable reovirus replication in the tumor tissues treated with both cetuximab and reovirus-activated NK cells, whereas the virus not detectable in tumors treated reovirus-activated NK cells alone (Fig. 3d, p < 0.001). These results suggested that cetuximab activity enabled viral replication and lysis in tumor cells, potentially by mediating viral transfer from NK cells to tumor cells.

**Fig. 4:**
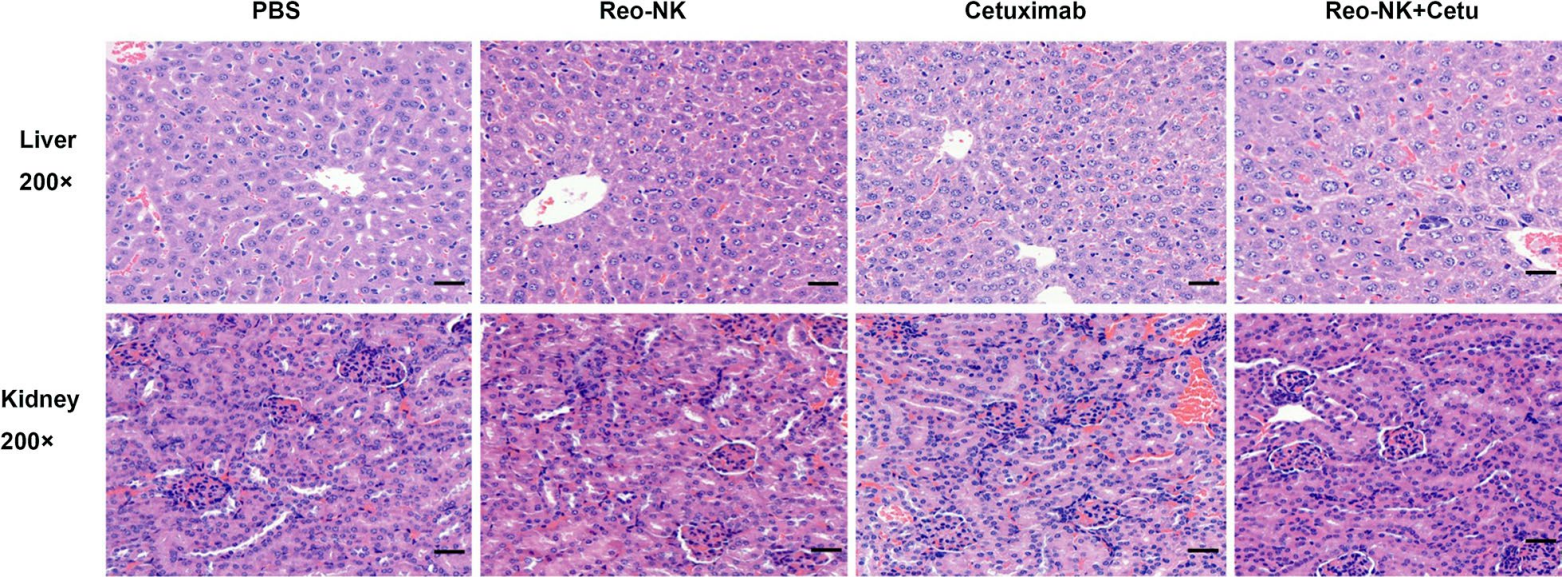
Representative H&E staining of liver and kidney from mice with different treatments. Scale bars, 50 μm

The potential side effects or toxicity of combination therapy are always a great concern in clinical application. The side effect study of reovirus-activated NK cell combined with cetuximab was conducted by H&E staining. Liver and kidney were collected on day 26 and subjected to H&E staining. The results indicated that no toxic pathological changes in mice’s liver and kidney (Fig. 4).

### TLR3 senses reovirus in NK cells

**Fig. 5:**
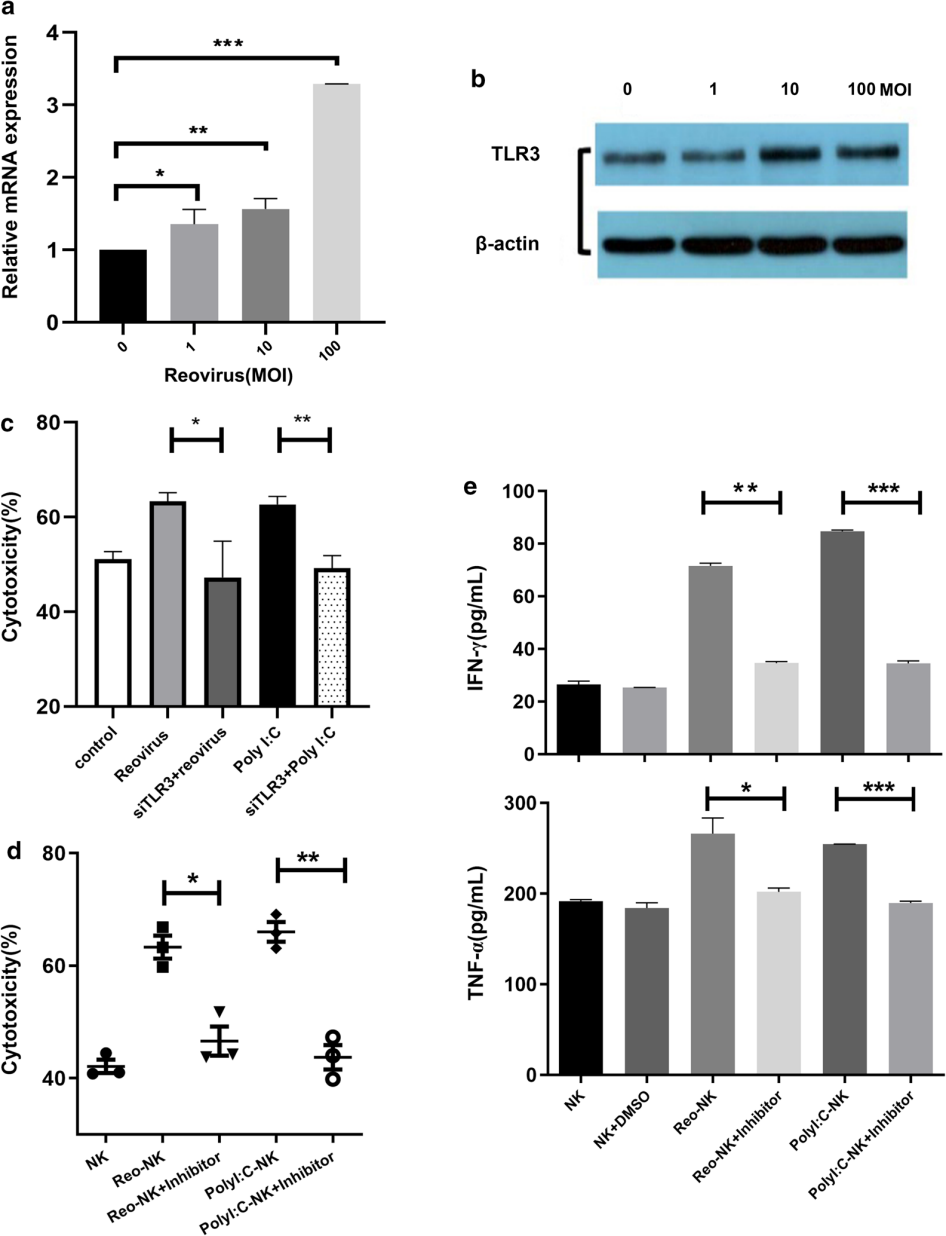
TLR3 is required for NK cell response to reovirus. NK cells were stimulated with 1, 10, or 100 MOI reovirus for 12 h. Untreated NK cells were used as a control. **a** Fold changes in TLR3 gene expression of NK cells relative to control cells, measured by qPCR. **b** Western blot analysis of TLR3 expression of NK cells. **c** NK cells were transfected with siTLR3, and knockdown NK cells were then stimulated with 10 MOI reovirus or 1 μg/mL Poly(I:C) for 12 h. Cytotoxicity of control and TLR3 knockdown NK cells against DLD-1 cells was measured by CCK-8 assay. **d** NK cells were activated with 10 MOI reovirus or 1 μg/mL Poly(I:C) in the presence or absence of 10 μM TLR3/dsRNA complex inhibitor for 12 h. Cytotoxicity of chemically inhibited and control NK cells against DLD-1 cells was measured by CCK-8 assay. **e** TNF-α and IFN-γ levels in culture supernatants were quantified by ELISA. Error bars represent means ± SD. *p < 0.05, **p < 0.01, ***p < 0.001

To examine the mechanisms by which reovirus enhances NK cell cytotoxicity.We first determined which receptor participated in reovirus-induced NK cell activation. Based on our previous evidence that NK cells were activated by reovirus in a mechanism apparently similar to that of Poly(I:C), we therefore focused on the reovirus dsRNA genome. TLR3 is anchored to the endosomal membrane to recognize extracytoplasmic dsRNA, such as Poly(I:C).We reasoned that reovirus could infect NK cells, thereby delivering dsRNA to the cell interior, and subsequently activating TLR3, similar to lipofectamine-packed Poly(I:C). To test this hypothesis, we first determined mRNA and protein expression levels of TLR3 in NK cells, and found that TLR3 mRNA and protein levels were both elevated in NK cells after reovirus administration (Fig. 5a and b). The expression of TLR3 was also confirmed by intracellular staining and flow cytometry, which revealed a slight increase in TLR3 in reovirus-treated NK cells (Additional file 2: Figure S1), a finding which was also consistent with the results of Western blotting.

To further confirm that TLR3 may participate in reovirus-induced NK-cell activation, we used siRNA targeting to knockdown TLR3 expression in reovirus-activated NK cells (Additional file 3: Figure S2). We then found that TLR3 knockdown significantly reversed the reovirus- or Poly(I:C)-induced cytotoxicity (Fig. 5c), while negative control NK cells remained responsive to reovirus activation. We then used TLR3/dsRNA complex inhibitor, a competitive inhibitor of dsRNA binding to TLR3, to confirm the contribution of TLR3/dsRNA binding in reovirus-mediated NK cell activation. The results of this experiment showed that the inhibitor significantly decreased both reovirus- and Poly(I:C)-enhanced cytotoxicity (Fig. 5d). In addition, release of TNF-α and IFN-γ, well-established potent anti-tumor factors, was also significantly induced after 24 h of stimulation with 10 MOI reovirus or Poly(I:C), but decreased after treatment with TLR3/dsRNA complex inhibitor (Fig. 5e).Together, these results indicate that TLR3 serves as the primary receptor for modulating the activation and function of NK cells by reovirus.

### TBK/IKKε mediates NK cell response to reovirus

**Fig. 6:**
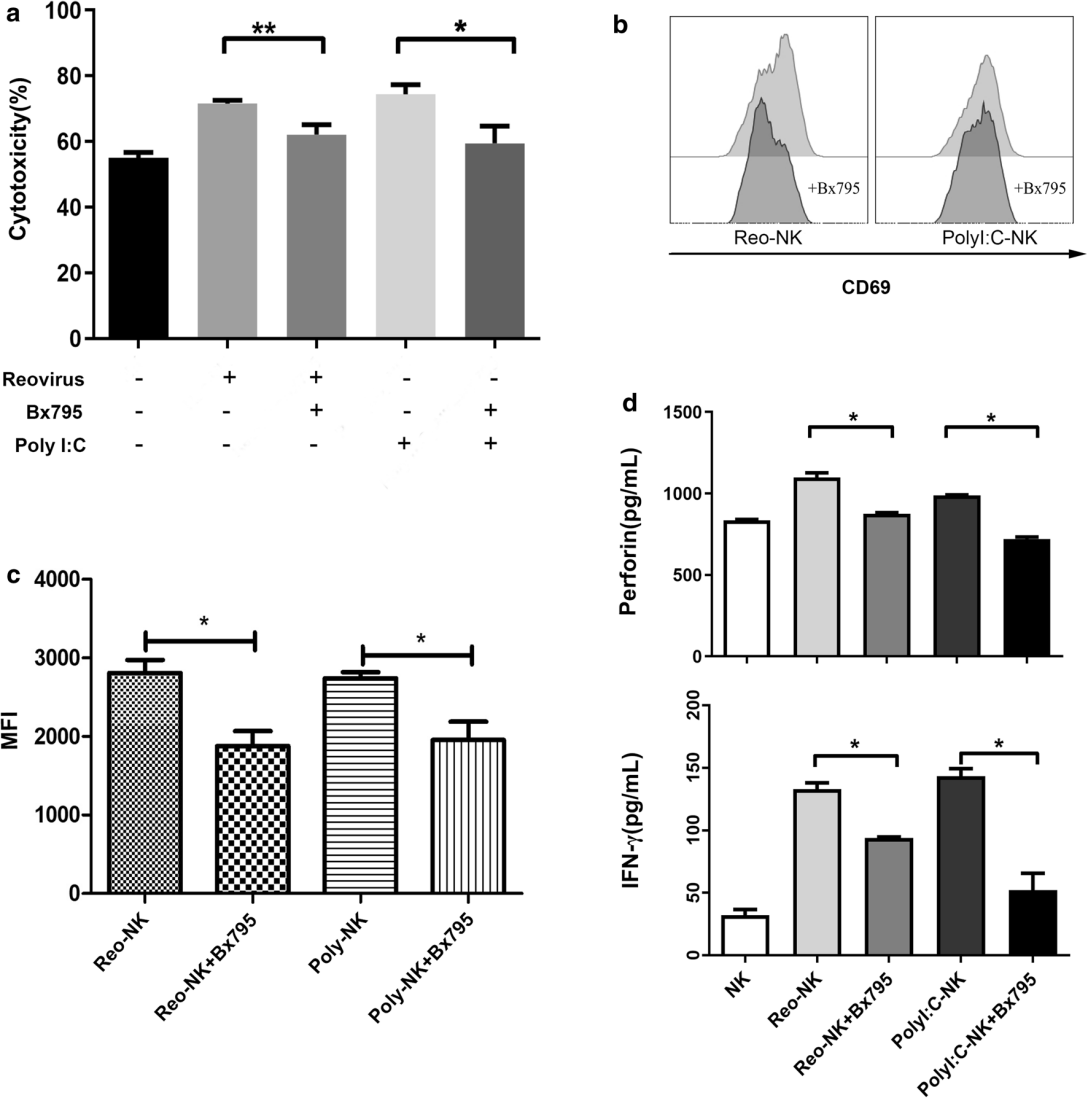
TBK1/IKKε mediates NK cell response to reovirus. **a** NK cells were activated with 10 MOI reovirus or 1 μg/mL Poly (I:C) in the presence or absence of TBK1/IKKε inhibitor BX795 (10 μM) for 12 h, then co-cultured with DLD-1 cells at E:T ratio of 5:1 for 4 h. Cytotoxicity was measured by CCK-8 assays. Data represent means ± SD. **b-c** Representative histograms of CD69 expression and the fold increase in MFI for CD69 in NK cells determined by flow cytometry **d** Perforin and IFN-γ in the supernatants were quantified by ELISA. *p < 0.05, **p < 0.01.and the fold increase in MFI for CD69

To further clarify the pathway for TLR3-dependent reovirus activation of NK cells, we examined the effect of blocking TBK1/IKKε, a downstream effector molecule in the TLR3 signaling pathway. To this end, NK cells were incubated for 12 h with 10 MOI reovirus in the presence of Bx795, a small molecule inhibitor of TBK1/IKKε. We found that Bx795 inhibited reovirus and Poly(I:C) enhancement of NK cell cytotoxicity (Fig. 6a). In addition, CD69 expression on NK cells was similarly decreased after Bx795 treatment (Fig. 6b, c). Furthermore, we observed that perforin secretion was increased with exposure to 10 MOI reovirus or 1 μg/mL Poly(I:C), but decreased with Bx795 treatment, and that IFN-γ secretion from NK cells was also reduced after TBK1/IKKε blockade (Fig. 6d). These results cumulatively show that TLR3-dependent response to reovirus is mediated by the TBK1/IKKε signaling.

## Discussion

Oncolytic viruses (OV) are a promising class of anti-cancer therapeutics that selectively infect, replicate within, and kill tumor cells. The unique susceptibility of cancer cells to OV infection is the result of defective immune responses and aberrant cellular signaling that accompanies tumorigenesis [27–29]. Following internalization, oncolytic viruses hijack the cell’s transcriptional and translational machinery and trigger cell death by a variety of necrotic, apoptotic, and immune-mediated pathways. Reovirus is a nonenveloped dsRNA virus that preferentially replicates in *KRAS* mutant cells. In light of this interesting and potentially extremely useful property of specifically targeting and killing tumor cells, reovirus has thus come under evaluation as a therapeutic agent in many clinical trials, worldwide [30, 31]. For example, a phase I CRC clinical trial is currently underway to test the efficacy of oncolytic reovirus in combination with chemotherapy (NCT01274624) [32]. In addition to direct oncolysis, recent discoveries have also shown that antigens released from reovirus-infected tumor cells are capable of inducing potent anti-tumor immunity, which may be pivotal to the therapeutic effects of the virus [33–35].

In this study, we demonstrate that ex vivo-expanded NK cells can be directly activated by reovirus. NK cells activated by reovirus showed strongly enhanced cytotoxicity against different human CRC cell lines, regardless of differing levels of EGFR expression and *KRAS* mutation status. In addition, exposure to reovirus led to significant up-regulation of the NK activation marker CD69 as well as transcriptional induction of perforin, granzymes, and TNF. Previous studies revealed that reovirus preferentially replicates in and induces apoptosis of *KRAS* mutant CRCs, and that cetuximab-mediated ADCC activity is correlated with the expression level of EGFR. Wild-type *KRAS* or low EGFR expressing tumor cells are therefore likely to exhibit increased resistance to lysis by reovirus or cetuximab-mediated ADCC [6, 36, 37]. In this study, we observed that *KRAS*-mutant and *KRAS*-WT CRC cell lines showed similar sensitivity to reovirus-activated NK cell killing, and furthermore, tumor cells with both high and low EGFR expression were equally sensitive to cetuximab-induced ADCC, thus overcoming these obstacles to monotherapy. Reovirus activation of NK cells in combination with cetuximab thus provides a versatile strategy to circumvent tumor resistance that can be modified to overcome tumor resistance to other tumor-targeting therapies.

**Fig. 7:**
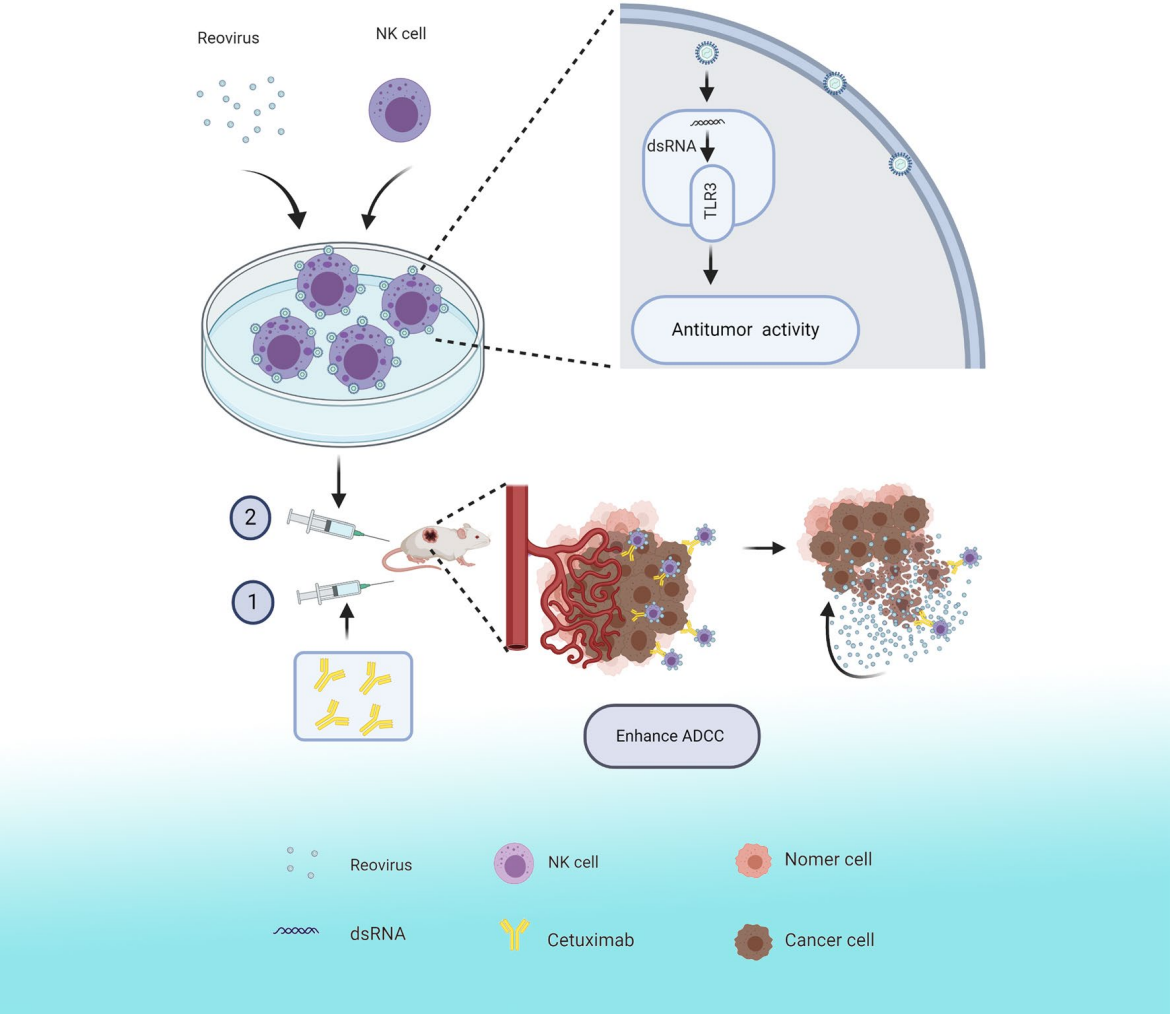
Schematic illustration

As mentioned above, reovirus is currently under evaluation as an oncolytic agent in numerous clinical trials. Unfortunately, reovirus is ubiquitous in the environment and most adults possess NABs due to prior exposure or following intravenous therapy [38]. Thus, reovirus therapy is seriously curtailed by NABs if administered systemically. However, previous work has revealed that reovirus can be protected from NABs by carriage on PBMCs or DCs, which can also deliver reovirus to the tumor site [39]. Therefore, combination of reovirus with cytotoxic adaptive cell therapy shows considerable promise as an anticancer therapeutic strategy [40, 41]. In previous work, we have shown that cytokine-induced killer (CIK) cells or ex vivo-expanded NK cells can be used as a protective delivery vehicle to carry reovirus to the tumor, avoiding antibody neutralization [21, 42]. Moreover, cytotoxicity of CIK cells was increased after loading with reovirus [43]. In this study, the combination strategy can be summarized as follows(Fig. 7):ex vivo-expanded NK cells were pretreated and loaded with reovirus, which subsequently activated the NK cells and enhanced their cytotoxicity. Our data further show that reovirus-loaded NK cells are capable of depositing active reovirus onto tumor sites when combined with cetuximab. Once in contact with reovirus-loaded NK cells, the cetuximab potentially functions as a bridge to facilitate delivery of the reovirus to the tumor cells.

Although previous in vitro studies have shown that NK cells can be activated by reovirus within PBMCs, resulting in increased NK anti-leukemia activity, which suggests that reovirus in fact activates DCs or monocytes, which in turn activate the NK cells [44, 45]. Moreover, results from a colorectal cancer clinical trial also suggested that a reovirus-mediated increase in human NK cell activity in vivo was likely due to cross-talk between NK cells and reovirus-activated DCs [46]. This discrepancy with our data is likely due to differences in experimental conditions. First, we used highly purified, fresh, ex vivo-expanded NK cells (> 95%) instead of PBMCs. Second, we stimulated NK cells with different titers of reovirus, and found reovirus stimulation enhanced NK cell cytotoxicity in a dose-dependent manner. Finally, the cytotoxicity of reovirus-activated NK cells was reduced after TLR3 knockdown or blockade of TLR3 signaling pathways and, we presume, the mechanism of TLR3-dependent NK cell activation by reovirus.

TLR3 is a pattern recognition receptor that recognizes and responds to dsRNA. We found only moderate levels of TLR3 expression in unstimulated NK cells, in agreement with results reported by Schmidt et al. [24]. The use of TLR3 agonists to enhance the cytotoxicity of NK cells has been well-described [47, 48]. Poly(I:C) is a synthetic dsRNA analog which has been consistently used to induce TLR3 signaling in vitro and in vivo. Previous studies have shown that NK cells can directly recognize and respond to Poly(I:C),which stimulates NK cell-mediated cytotoxicity and leads to the up-regulation of TLR3 and activation marker CD69 [24]. NK cells respond to Poly(I:C) by producing IFN-γ. In this study, we confirmed that the cytotoxicity of NK cells was enhanced by reovirus, and we show that stimulation with reovirus increased TLR3 transcript and protein levels in NK cells. Furthermore, our results demonstrate that TLR3 knockdown significantly reduced reovirus-activated NK cell cytotoxicity, and that inhibitor of TLR3/dsRNA complex, which prevents dsRNA binding to TLR3, also significantly reduced reovirus activation of NK cell cytotoxicity and cytokine (TNF-α and IFN-γ) release. Consistent with these results, we show that in reovirus-activated NK cells, cytotoxicity, CD69 expression, and perforin and IFN-γ secretion were all reduced by treatment with Bx795, an inhibitor of TBK1/IKKε that acts as a common mediator in the signaling pathways of TLR3. Poly(I:C) was used in these experiments as a positive control. These results thus show that TLR3 is critical for NK cell response to Poly(I:C) and reovirus, and that reovirus promotes the activation of NK cells through a mechanism that is probably similar to that of Poly(I:C).

## Conclusions

In summary, this study provides insight into ex vivo-expanded NK cell activation by reovirus for CRC therapy. We demonstrated that reovirus directly enhances NK cell cytotoxicity against CRC cells in a TLR-3-dependent manner. NK cells act as carriers to transfer the reovirus to the tumor site, and the combination of cetuximab and reovirus-activated NK cells enhances the ADCC effect. As a result, reovirus can directly lyse tumor cells at the tumor site, while reovirus activated-NK cells enhance the ADCC effect to kill tumors, thus providing a framework for further development of effective, clinical strategies against CRC.

## Supplementary Information

**Additional file 1: Table S1.** The PCR primers used in this study.

**Additional file 2: Figure S1.** Expression of intracellular TLR3 in reovirus-treated NK cells.

**Additional file 3: Figure S2.** Western blots showing TLR3 expression in NK cells transfected with siTLR3.

## Data Availability

The datasets analyzed during the current study are available from the corresponding author on reasonable request.

## Abbreviations

CRC: Colorectal cancer
NABs: Neuralizing antibodies
EGFR: Epidermal growth factor receptor
ADCC: Antibody-dependent cell-mediated cytotoxicity
DCs: Dendritic cells
NK: Natural killer
TLR3: Toll-like receptor 3
Poly (I:C): Polyinosinic:polycytidylic acid
PBMCs: Peripheral blood mononuclear cells
CCK-8: Cell Counting Kit-8
dsRNA: Double stranded RNA
MOI: Multiplicity of infection
PFU: Plaque-forming unit
ACT: Adoptive cell therapy
CIK: Cytokine-induced killer
