# Supplementary material for: Reovirus enhances cytotoxicity of natural killer cells against colorectal cancer via TLR3 pathway
Source: J Transl Med. 2021 May 1;19:185. doi: 10.1186/s12967-021-02853-y (PMC8088708; doi:10.1186/s12967-021-02853-y)
Supplement: Supplementary file 1 — Additional file 1: Table S1. The PCR primers used in this study. [file 12967_2021_2853_MOESM1_ESM.docx]

Table 1. *Primers used for PCR*

| Gene Name |  | Sequences(5‘→3‘) Size of Amplicon(bp) | |
| --- | --- | --- | --- |
| $\beta$-actin |  | | |
| Forward | GCCGGGACCTGACTGACTAC | | 100 |
| Reverse | TTCTCCTTAATGTCACGCACGAT | |  |
| GZMH |  | |  |
| Forward | CTGGCTGGGGTTATGTCTCAA | | 203 |
| Reverse | GGCTACGTCCTTACACACGAG | |  |
| GZMM |  | |  |
| Forward | ACACCCGCATGTGTAACAACA | | 196 |
| Reverse | GGAGGCTTGAAGATGTCAGTG | |  |
| PRF1 |  | |  |
| Forward | CCCTCTGTGAAAATGCCCTAC | | 116 |
| Reverse | GCTGACTTTGGCCCTGGTTA | |  |
| TNF |  | |  |
| Forward | TCCAGGCGGTGCTTGTTC | | 142 |
| Reverse | CCAGAGGGCTGATTAGAGAG | |  |
| T3D |  | |  |
| Forward | TGATTTCCATTACTTCTGCTGCTT | | 72 |
| Reverse | TCCTGTTCACGATTCCATCAGAT | |  |
| TLR3 |  | | |
| Forward | TAGCAGTCATCCAACAGAATC | | 85 |
| Reverse | CACACAGCATCCCAAAGG | |  |
