# Supplementary figures and images for: Reovirus enhances cytotoxicity of natural killer cells against colorectal cancer via TLR3 pathway
Source: J Transl Med. 2021 May 1;19:185. doi: 10.1186/s12967-021-02853-y (PMC8088708; doi:10.1186/s12967-021-02853-y)

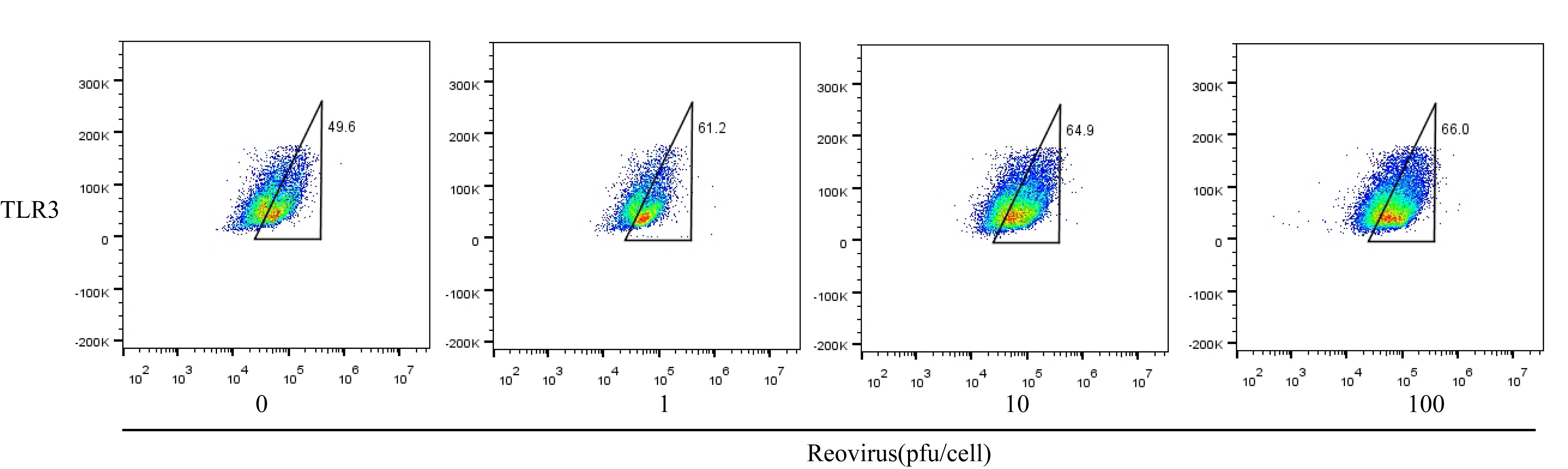

Supplement: Supplementary file 2 — Additional file 2: Figure S1. Expression of intracellular TLR3 in reovirus-treated NK cells. [file 12967_2021_2853_MOESM2_ESM.jpeg]

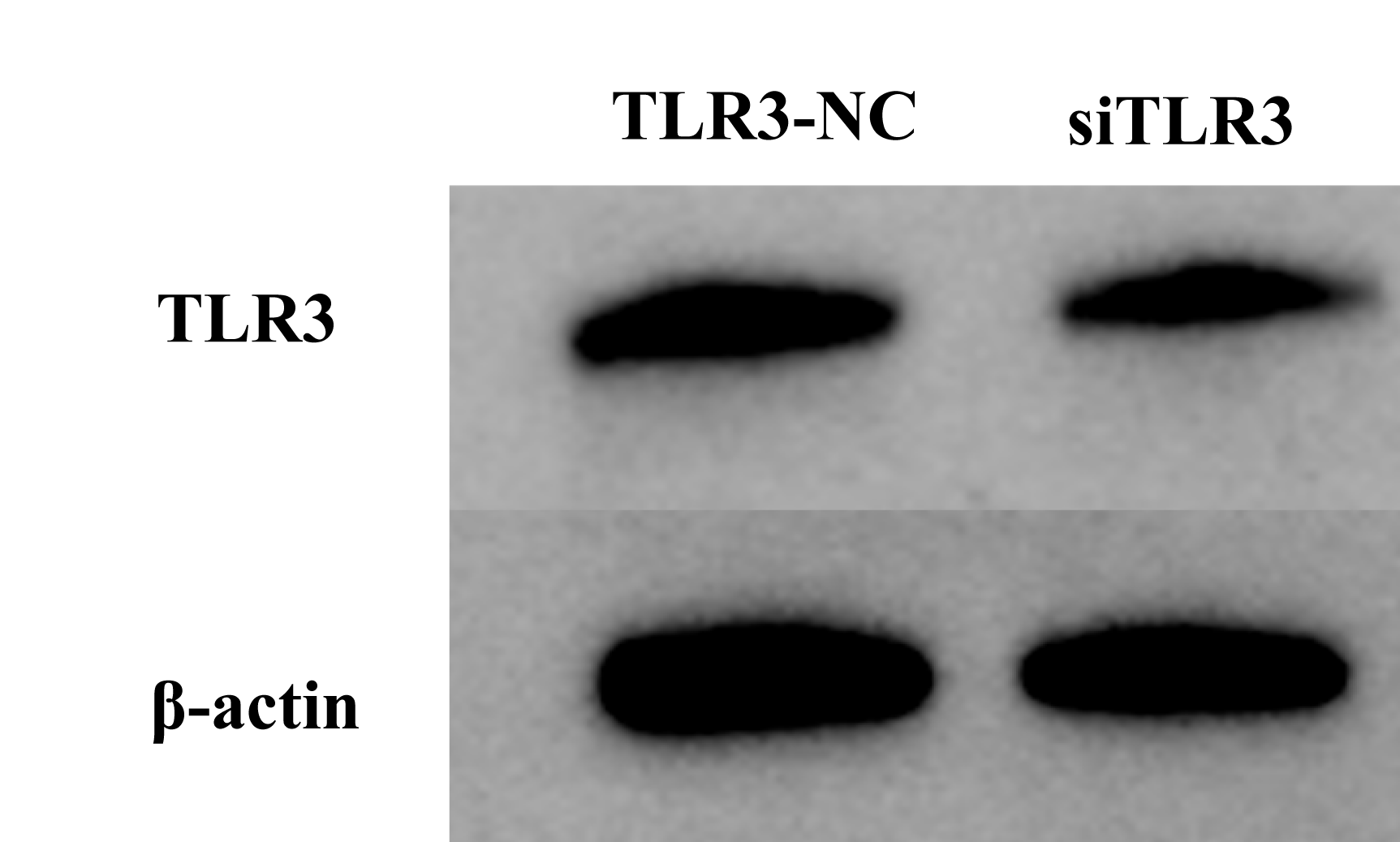

Supplement: Supplementary file 3 — Additional file 3: Figure S2. Western blots showing TLR3 expression in NK cells transfected with siTLR3. [file 12967_2021_2853_MOESM3_ESM.tiff]
